# Insights and Recommendations From Two Urology-Based Educational Interventions: Development of a Urology Teaching Programme and Out-of-Hours Urology Handbook

**DOI:** 10.7759/cureus.46849

**Published:** 2023-10-11

**Authors:** Saakshi Bansal, Jimena Alvarez del Castillo Gonzalez, Yanjinlkham Chuluunbaatar, Andrew Brodie

**Affiliations:** 1 Department of Urology, Lister Hospital, Stevenage, GBR; 2 Department of Colorectal Surgery, The Royal Marsden Hospital, London, GBR

**Keywords:** quality improvement project, online teaching programme, urology handbook, urology, medical education

## Abstract

Exposure and education in urology for medical students (MSs) and junior doctors (JDs) have long been overlooked, resulting in inadequate preparedness for the management of urological cases/patients. This study addresses this deficiency through the implementation of a Urology Teaching Programme (UTP) and the creation of an Out-of-Hours Urology Handbook (OOHUH). The UTP was delivered virtually, targeting senior MSs and JDs, and covered common urological presentations and management pathways from a JD's perspective. The OOHUH aimed to enhance the care provided by general surgery senior house officers (SHOs) at Lister Hospital (Stevenage, UK), offering guidance for nine common urological conditions in emergency and out-of-hours settings. Both initiatives demonstrated significant improvements in knowledge and confidence in urology. The findings underscore the importance of supplementary urology education and suggest strategies for bridging training gaps in medical curricula and clinical practice. Recommendations include tailored induction programs and simulation days for junior doctors, along with the widespread adoption of such educational interventions to enhance patient care and trainee preparedness in urology.

## Introduction

Exposure and education in urology for medical students (MSs) and junior doctors (JDs) are often overlooked [1-7]. Various research has revealed that an average of only 50% of medical students have a mandatory urology rotation during medical school, which only lasts for one week on average, despite the common occurrence of urology rotations in their early practice years [7,8]. Between 20-30% of JDs have a urology rotation lasting four months, yet studies consistently reveal that juniors feel unprepared for urological cases and procedures [6,9,10]. Moreover, confidence in managing urological issues is vital not only during dedicated urology rotations, but also in scenarios like primary care, cross-covering surgical specialties out-of-hours (OOH), and handling urological problems in general medicine; approximately 25% of all surgical referrals in hospital are urological, and urology cases form around 10% of general practitioners (GP) consultations [11-14]. Furthermore, there is a lack of research in urology education, hindering the identification of areas requiring improvement [15].

It has been demonstrated that tailoring medical educational curricula positively impacts trainees when learning core medical skills. However, the process of modifying curricula is often lengthy and frustrated by multiple obstacles [16-19]. As an alternative, supplementary courses can offer the necessary knowledge to medical students and overcome the challenges of extensive curriculum changes; these courses can effectively address gaps in medical training [10,20,21]. Online platforms in particular have gained prominence, especially since the COVID-19 pandemic, for delivering such supplementary education [21-23].

Having faced personal challenges with urological cases and hearing similar accounts from colleagues, largely due to inadequate training, we created a urology teaching programme (UTP). The UTP was aimed at senior MDs and JDs, and the topics covered common urological presentations with the management pathways from a JD’s perspective.

Additionally, we conducted a quality improvement project (QIP) and crafted an out-of-hours urology handbook (OOHUH) outlining investigation and management protocols for nine common urological conditions seen in emergency and after-hours settings. The handbook targeted general surgery SHOs (senior house officers), ranging from foundation year 2 (FY2) to core training 2 (CT2) level JDs, responsible for covering urology OOH. This took place at Lister Hospital, Stevenage, where general surgery SHOs are first-on-call and accept the referrals for the urology take OOH, with remote back-up from a urology registrar as second-on-call. Registrars offer guidance via phone and are required to attend in person if urgent procedures or complex cases arise.

## Materials and methods

Urology teaching programme

Creating and delivering the UTP consisted of several steps: 1) selecting session topics, 2) choosing the mode of delivery, 3) advertising to hospitals and universities in other regions, and 4) finalising course timing/length.

The majority of the team, including junior members, was based at Lister Hospital, Stevenage. The UTP was directly supervised by a senior urology registrar and overseen by a consultant. Initially, four weekly sessions were held over a month for multiple reasons: 1) the programme was new and not established; 2) the course had broad coverage of the specialty for JD relevance without covering complex detail; 3) time constraints.

The pilot session covered "Catheters," an essential topic for urology juniors, focusing on types of commonly used catheters and the management of complications. Subsequent topics were determined by participant voting via the feedback forms. Consequently, the four topics for the UTP were: 1) catheters, 2) ureteric colic, 3) the acutely painful and swollen scrotum, and 4) urological imaging.

Virtual delivery was chosen using "Microsoft Teams®" to include participants from different regions. The promotion involved liaising with foundation programme teaching organizers, university society presidents, and local participants for dissemination. After the first session, a sign-up sheet was established, collecting interested participants' email addresses for a mailing list.

Sessions commenced on 04/05/23 at 1800 hrs and continued each Thursday until 25/05/23. Feedback forms were gathered, attendance tracked, and recordings shared for those unable to attend live.

Out-of-hours urology handbook quality improvement project

To enhance the care provided by general surgery SHOs covering the urology take OOH, we first established which areas required improvement. Questionnaires were distributed to the urology second-on-calls (registrars) from January 23 to March 23, providing feedback on OOH cover to pinpoint potential enhancements. Starting April 23, we gathered data from the incoming and existing general surgery SHOs, evaluating their confidence in covering urology.

Using this feedback, an OOH urology handbook was created, under the supervision of a senior urology registrar. Approved by the Clinical Director of Urology at Lister Hospital, the handbook addresses nine common urological presentations and management. These presentations include “bypassing catheter”, “urinary retention/anuria”, “difficult catheterisation”, “renal/ureteric calculi”, “haematuria”, “pain/haematuria post stent insertion”, “pyelonephritis/UTI”, “paraphimosis”, and “testicular/scrotal pain”. To assess the impact, we collected second-cycle data from questionnaires between April 23 and June 23, reviewing the handbook’s influence on both participant groups.

## Results

Urology teaching programme

Fourty-two MSs/JDs or equivalent joined the UTP mailing list. Sessions had an average of 12 participants, with participant numbers increasing in the latter sessions. Attendees originated from several different regions, including Cambridgeshire, Hertfordshire, Buckinghamshire, Glasgow, and London, and one participant from Mexico. We gathered 29 feedback forms across the four weeks, reflecting an overall positive response to the teaching. Descriptive and thematic analyses were chosen to examine the data more carefully.

**Figure 1:**
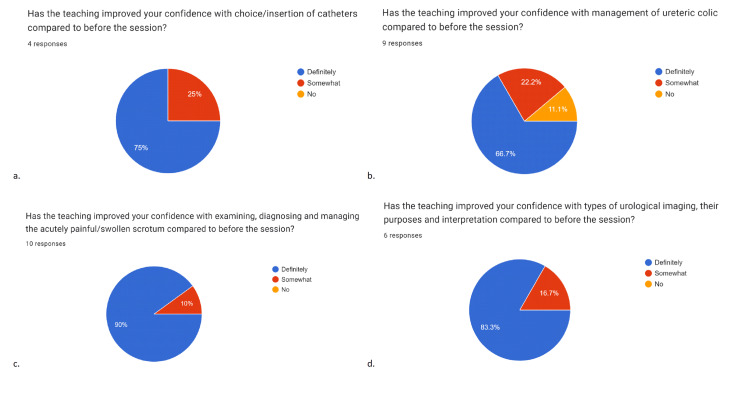
Data collated from feedback forms Data has been represented in pie charts created from feedback forms after each teaching session (a-d), showing values in percentages (%). Twenty-nine responses were collected over four sessions in total. All charts show that confidence in the topic covered had improved for all or most participants post-session. Figures 1a, 1b, 1c, 1d depict an increase in confidence in the management of catheters in 100% (n=4), management of ureteric colic in 88.9% (n=9), management of scrotal pain in 100% (n=10), and urological imaging in 100% (n=6) of participants respectively. 78.8±9.9% (CI=95%, n=29) on average (mean value) felt "definitely" more confident on the topic covered. Data is represented as absolute percentages and mean±SEM. CI = confidence interval, SEM = standard error of the mean.

**Figure 2:**
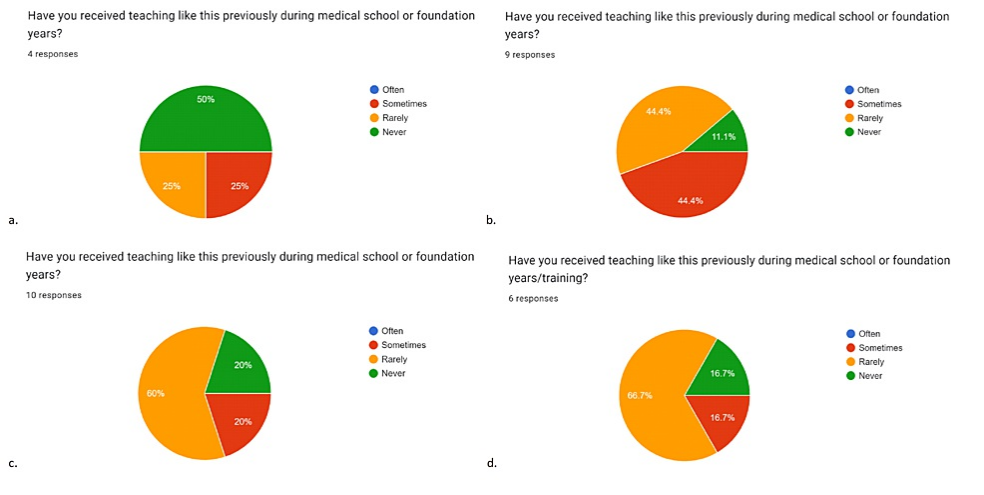
Data created from feedback forms after each teaching session Data has been represented in pie-charts created from feedback forms after each teaching session (a-d; a=catheters, b=ureteric colic, c=acutely painful and swollen scrotum, d=urological imaging), showing values in percentages (%). Twenty-nine responses were collected over four sessions in total. Charts suggest most participants had either “rarely” or “never” previously received teaching on the covered topics. Figures 1a, 1b, 1c, 1d depict that 75% (n=4), 55.5% (n=9), 80% (n=10) and 83.4% (n=6), respectively, had either “rarely” or “never” received similar teaching on the topics covered; mean value = 73.5 ±10.6% (CI=95%, n=29). Data represented as absolute percentages and mean±SEM. CI = confidence interval, SEM = standard error of the mean.

On average (mean values), 79% (n=29) of participants reported that they definitely felt more confident in the topic covered during the session, 98% learned something new, and 100% found the teaching was aimed at the appropriate level. Additionally, 93% (n=29) anticipate an improvement in clinical practice as a result of the teaching. Notably, 73% (n=29) reported that they had either “never” or “rarely” received similar teaching during their prior medical education. Results are summarised in Figures 1-2 below.

The thematic analysis assessed qualitative responses for two questions: “please elaborate on things you learnt today that you didn't know before, if anything?” and “please suggest any improvements for the session, if any.” The themes emerging from the first question were categorised into the following: enhanced practical skills, accuracy in diagnosis and insights into management, interpreting medical investigations, and knowing when to escalate to seniors. The themes from the second question were categorised into the following: no improvements suggested, the inclusion of questions to test knowledge, the inclusion of pathophysiology, expanding the range/number of topics/sessions, and removing technicalities irrelevant to JDs.

Feedback included remarks such as “I'm now much clearer on how to manage ureteric colic as a junior, and the worrying features that would cause you to escalate”, “I learned to perform the procedure of insertion of catheters, likewise the care that must be taken at the time of performing them”, and “it was really helpful to go through information relevant for junior doctors”.

Out-of-hours urology handbook quality improvement project

For the QIP, the initial data collection from eight incoming and existing general surgery SHOs indicated limited confidence in urology management and significant role-related concerns. Regarding confidence in handling the nine aforementioned urological conditions, the average (mean) confidence level was 4.2/9 (47%, n=8). Around 87.5% (7/8) were uncertain about which patients require overnight emergency surgery, 87.5% (7/8) felt unsure about the required steps to take before calling the on-call registrar, and 50% (4/8) lacked clarity on handover content. 

Analysis of responses to the question, “do you have any concerns about covering urology OOH? If yes, please elaborate?” revealed the following concerns: over-admission or improper discharges, uncertainty of when to escalate, unclear referral/follow-up pathways, how to contact the registrar, and balancing general surgery and urology take concurrently.

**Figure 3:**
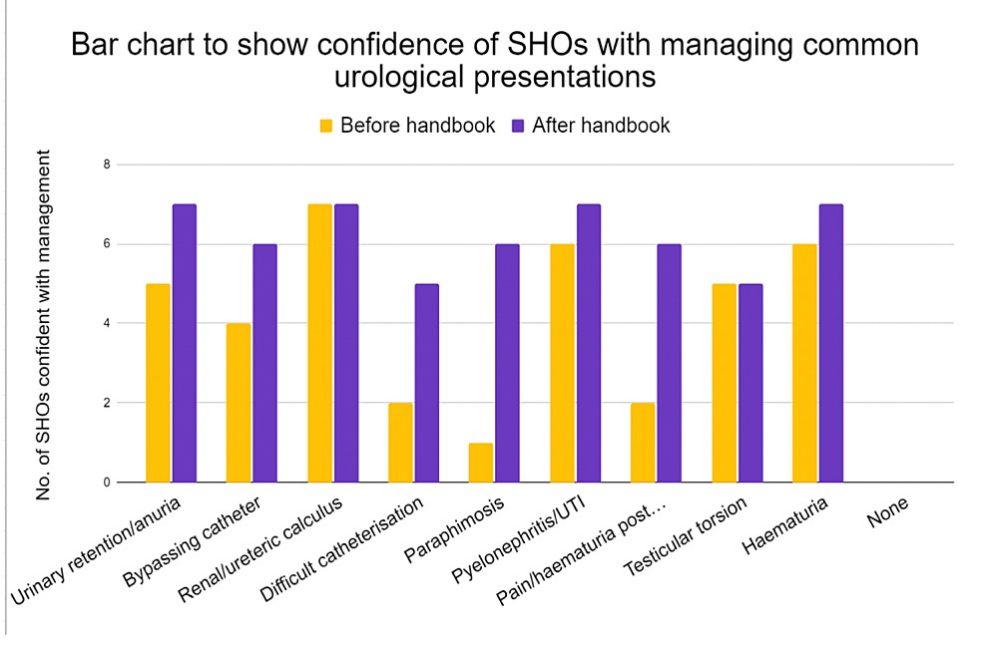
SHOs’ confidence in managing the nine urological conditions commonly presenting OOH Data has been represented in a double-bar chart demonstrating the considerable increase in senior house officers (SHOs’) confidence in managing the nine urological conditions commonly presenting OOH. Pre-handbook, the average number of senior house officers (SHOs) confident with managing the nine presentations was an average of 4.2/9 (47%, n=8), which improved to 6.2/9 (69%, n=7) post-handbook. Improvement was demonstrated in all presentations except “renal/ureteric calculus” and “testicular torsion” for which the majority were already confident. Averages calculated as absolute means.

**Figure 4:**
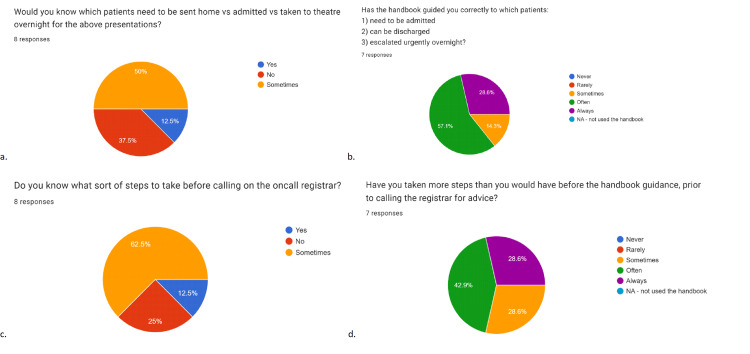
Representation of data created from cycle data collection from general surgery SHOs Data has been represented in pie charts created from the first (a and c) and second (b and d) cycle data collection from general surgery senior house officers (SHOs). Charts “a” and “b” demonstrate the increase in knowledge of which patients require emergency theatre overnight as per handbook guidance; 87.5% (n=8) were not always sure which patients require emergency theatre pre-handbook, whereas post-handbook, 85.7% (n=7) reported they were now “often” or “always” guided correctly by the handbook. Charts “c” and “d” show the increase in steps taken before escalating to the registrar due to handbook guidance; 87.5% (n=8) were not always sure of steps to take before escalating to the registrar, whereas post-handbook, 71.5% (n=7) reported “always” or “often” taking more steps prior to escalating due to handbook guidance. Data represented as absolute percentages (%).

Following the distribution of the urology handbook, the second cycle data collection from seven of the original eight general surgery SHOs yielded promising outcomes. Confidence in managing the nine urological conditions notably increased, with the mean now 6.2/9 (69%, n=7) (Figure 3). All SHOs (100%, n=7) utilised the urology handbook during their on-call shifts. 86% (6/7) reported being correctly guided by the handbook either “always” or “often” for discharging/admitting decisions, and 72% (5/7) took more preliminary steps before consulting the registrar. A unanimous 100% (7/7) reported overall increased confidence due to the urology handbook and the handbook received an average rating of 4.7/5 for usability. Results are summarised in Figures 3-4 below.

Regarding improvements to the handbook, most SHOs couldn’t suggest any. One proposed enhancing the discussion of complex presentations, but acknowledged the handbook's focus on manageable aspects for junior staff, with the expectation of complex cases being referred to the second-on-call. Feedback included statements such as “I think there is extensive information in the handbook about common presentations and I have felt fully prepared to see urology referrals overnight with the aid of the handbook”

Two out of seven (29%) SHOs in the second cycle reported that registrars had described a case of improper OOH patient discharge/admission. However, upon discussion, these SHOs clarified that the decisions leading to those scenarios were unrelated to the guidance in the handbook.

During initial data collection from registrars, 95% (n=21) of responses expressed a handbook would likely be useful. Qualitative data analysis indicated calls to registrars mostly concerned commonly assessed and managed urological issues. Handovers and assessment of urgency by SHOs required improvement. Quality of calls varied from adequate to needing significant improvement; examples of comments included “could be better”, “more clarity would have been nice but adequate quality commensurate with training level of the SHO”, “good summary, some basic assessment lacking”.

Fourteen out of 21 (67%) responses reported having to attend hospital during the on-call, five out of 14 (36%) of the reasons being for difficult catheterisations. Ten out of 21 (48%) responses noted that calls were largely for common presentations/cases suitable for SHO management; an example of a comment included being called for “common presentations (suprapubic catheter blocked but no bladder scans done yet etc)”.

**Figure 5:**
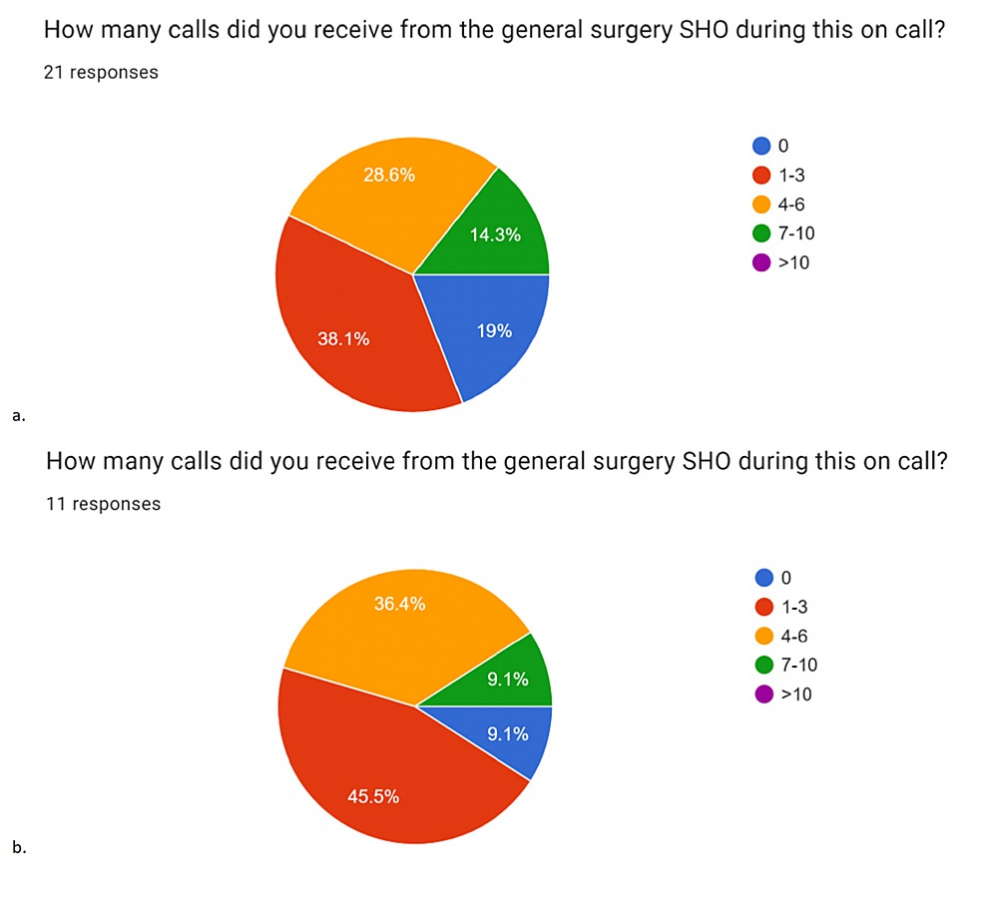
Data represented in pie charts created from cycle data collection from registrars Data represented in pie charts created from first (a) and second (b) cycle data collection from registrars. Charts “a” and “b” demonstrate that there was little change in the number of calls received by registrars OOH; the median number of calls received overnight by registrars in both cycles was 1-3 (first cycle n=21, second cycle n=11). Data in pie charts are represented as percentages (%) of responses falling into five different categories. Categories consist of a range of numbers to signify the number of calls received overnight: 0, 1-3, 4-6, 7-10, and >10.

**Figure 6:**
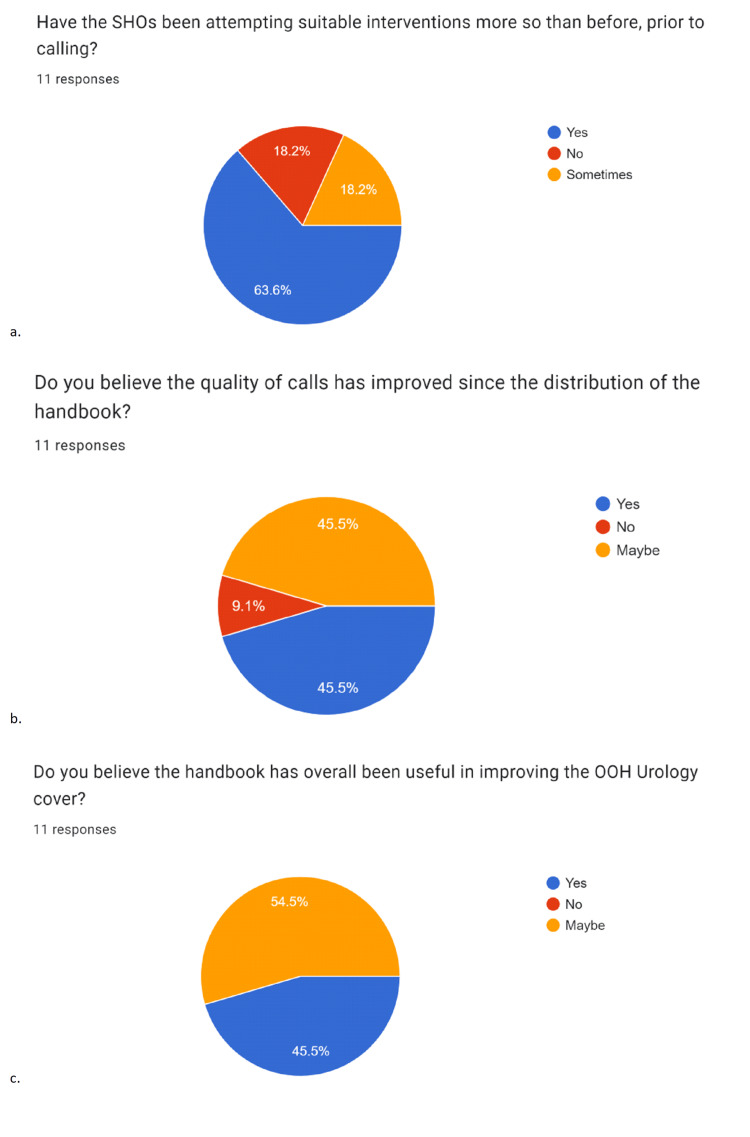
Data represented in pie charts created from second cycle data collection from registrars Chart “a” highlights positive registrar feedback, with 81.8% (n=11) responses reporting that senior house officers (SHOs) had been taking more steps prior to calling. Chart “b” shows that 90.9% (n=11) responses noted, to varying degrees, that the quality of calls had enhanced since the handbook, and 100% (n=11) agreed the handbook had been likely useful overall. Data represented as absolute percentages (%).

Second cycle data collection showed a similar frequency of escalation to specialist registrar (SpRs) OOH (Figure 5), but marked improvements in overall performance by SHOs (Figure 6). Responses revealed that 91% (n=11) noticed an improvement in the quality of calls, 82% (nine out of 11) noted an increase in the number of steps taken by SHOs prior to calling, and 91% (10/11) reported an improvement in handovers.

Qualitative data suggested that complex presentations dominated the calls in the second cycle; the most common (57%, n=11) reasons for registrar attendance were emergencies unfit for SHO management (e.g. emergency stenting). Notably, no registrars were called in for simple procedures like catheterisation.

Ten out of 11 (91%) responses noted that the calls were appropriate and required escalation; one case had been escalated despite being covered in the handbook. One response reported premature escalation - patients were not adequately assessed by the SHO beforehand. All participating SpRs found the handbook useful to varying degrees, positively impacting the cover overall.

## Discussion

Analysis and limitations of research

Numerous limitations surfaced during our data collection process, prompting ongoing adjustments to our approach. During planning, we recognized that the SHOs' previous urological experience could significantly impact their on-call confidence and introduce potential bias into our results. Consequently, we made the decision to exclude SHOs at the core-trainee level who had prior exposure to urology rotations, which often involves holding the registrar bleep for practical experience. Moreover, conducting the study in a single-centre setting impacts the generalisability of the results; however, the findings are in line with existing literature and only further solidify the requirement for enhanced urological teaching.

Another challenge encountered pertained to maintaining registrar continuity across the two data collection cycles. Over the six-month collection period, registrars exited the trust or left the on-call roster, with new second-on-calls introduced. This transition threatened the preclusion of direct comparison between SHO performances in the two cycles. Therefore, participant numbers had to be reduced to ensure that only the registrars involved in the first cycle gave feedback on the second one. Despite this, sufficient responses were amassed over the subsequent three months for thematic analysis.

Given the inherently subjective nature of confidence levels, individual variations can potentially impact result validity. However, we mitigated this concern by assessing the same SHOs' confidence levels pre- and post-handbook for identical presentations, thus minimising the influence of individual variability.

Completing questionnaires after demanding 24 or 48-hour shifts can introduce recall inaccuracies among registrars. At times, responses needed follow-up due to delayed completion, potentially misrepresenting the shift's quality due to the time elapsed between the shift and questionnaire submission.

The study on the urology handbook had a key limitation: it lacked a direct control group, making it challenging to accurately quantitatively assess the handbook's impact on confidence levels. This limitation stems from the natural increase in confidence over time as SHOs gain more experience. However, it's worth noting that registrars provided data over three months without the handbook in the first cycle, which was directly compared to their experiences with the handbook over another three-month period during the second cycle. This approach helped minimize the influence of the SHO experience on the results. Additionally, SHOs provided feedback before starting their rotation for the first cycle, and returned their second-cycle questionnaires within the first month of data collection, further reducing the impact of experience on confidence.

Learning points and recommendations

Key insights emerge from both studies, revealing consistent themes. A pivotal conclusion emerges: medical school and junior doctor education inadequately prepare junior doctors for urology-related tasks. Feedback from teaching sessions highlights that topics were rarely covered previously, while the handbook QIP demonstrates the significant enhancement achievable through a straightforward intervention like a basic handbook. Consequently, the integration of additional teaching on crucial topics seems feasible without undue disruption to existing curricula. This expansion promises enhanced clinical performance and patient care, potentially outweighing the challenges of curricular adjustment.

Nonetheless, as discussed in the introduction, obstacles to curriculum enrichment may deter such additions despite their potential merits. One strategy to surmount this hurdle involves an enhanced induction period for junior doctors on general surgery/urology rotations. Tailored and extended induction programs, guided by junior doctors' input on content, have shown positive effects on confidence with clinical management and preparedness [24-26]. Likewise, supplementary courses or simulation days emulating on-call scenarios can bolster junior doctors' confidence without compromising established teaching structures [27]. Notably, sessions on practical procedures and clinical skills, which are critical components of a surgical job, significantly enhance junior doctors' learning [20,24].

Disseminating and introducing the handbook to urology and general surgery departments presented challenges. Communication with both senior and junior members necessitated a multi-faceted approach. To achieve this, the handbook was presented at the general surgery departmental meeting to maximise reach. Additionally, the Clinical Director of Urology introduced the handbook and QIP during the urology departmental meeting, effectively reaching the registrars. Email distribution to junior doctors proved an efficient method for delivering the handbook to the intended audience.

Initial UTP promotion involved coordination with teaching organisation staff and medical student society leaders to spread awareness. However, this approach required constant second-hand communication; therefore, we introduced a sign-up sheet from the second session onwards to streamline communication. We shared the sheet link via initially established channels, formed a mailing list from sign-ups (43 in total), and directly corresponded with participants for subsequent interactions.

Furthermore, feedback from colleagues unable to attend sessions due to overlapping shifts prompted the decision to record sessions from the second session onward. Recordings were subsequently emailed to the mailing list, enabling participants to access the content retrospectively.

## Conclusions

It is evident that educational programs during both medical school and junior doctor training have not effectively prepared junior doctors for roles involving urology. This deficiency in urological exposure could impact patient care and potentially influence doctors' inclination to pursue urology training. However, swift and straightforward measures can address and bridge these training gaps, yielding beneficial outcomes. In light of this, we propose that medical schools and NHS trusts promptly adopt these modifications to enhance urological patient care while concurrently elevating junior doctors' confidence and experience within pertinent rotations.
